# Heterogeneity of Antibiotics Multidrug-Resistance Profile of Uropathogens in Romanian Population

**DOI:** 10.3390/antibiotics10050523

**Published:** 2021-05-02

**Authors:** Răzvan-Cosmin Petca, Silvius Negoiță, Cristian Mareș, Aida Petca, Răzvan-Ionuț Popescu, Călin Bogdan Chibelean

**Affiliations:** 1 “Carol Davila” University of Medicine and Pharmacy, 8 Eroii Sanitari Blvd., 050474 Bucharest, Romania; drpetca@gmail.com (R.-C.P.); silvius.negoita@umfcd.ro (S.N.); dr.razvanp@gmail.com (R.-I.P.); 2 Department of Urology, “Prof. Dr. Th. Burghele” Clinical Hospital, 20 Panduri Str., 050659 Bucharest, Romania; 3 Department of Anesthesiology and Critical Care, Elias University Hospital, 17 Marasti Blvd., 011461 Bucharest, Romania; 4 Department of Obstetrics and Gynecology, Elias University Hospital, 17 Marasti Blvd., 011461 Bucharest, Romania; 5 George Emil Palade University of Medicine, Pharmacy, Science, and Technology of Targu-Mures, 38 Gheorghe Marinescu Str., 540139 Targu-Mures, Romania; calin.chibelean@umfst.ro; 6 Department of Urology, Mureș County Hospital, 1st Gheorghe Marinescu Str., 540136 Targu-Mures, Romania

**Keywords:** urinary tract infections, UTIs, MDR, *Escherichia coli*, *Klebsiella*, uropathogens, AMR, antibiotic resistance

## Abstract

Urinary tract infections (UTIs) are a leading cause of morbidity for both males and females. The overconsumption of antibiotics in general medicine, veterinary, or agriculture has led to a spike in drug-resistant microorganisms; obtaining standardized results is imposed by standard definitions for various categories of drug-resistant bacteria—such as multiple-drug resistant (MDR), extensive drug-resistant (XDR), and pan drug-resistant (PDR). This retrospective study conducted in three university teaching hospitals in Romania has analyzed urine probes from 15,231 patients, of which 698 (4.58%) presented multidrug-resistant strains. *Escherichia coli* was the leading uropathogen 283 (40.54%), presenting the highest resistance to quinolones (R = 72.08%) and penicillin (R = 66.78%) with the most important patterns of resistance for penicillin, sulfonamides, and quinolones (12.01%) and aminoglycosides, aztreonam, cephalosporins, and quinolones (9.89%). *Klebsiella* spp. followed—260 (37.24%) with the highest resistance to amoxicillin-clavulanate (R = 94.61%) and cephalosporins (R = 94.23%); the leading patterns were observed for aminoglycosides, aminopenicillins + β-lactams inhibitor, sulfonamides, and cephalosporins (12.69%) and aminoglycosides, aztreonam, cephalosporins, quinolones (9.23%). The insufficient research of MDR strains on the Romanian population is promoting these findings as an important tool for any clinician treating MDR-UTIs.

## 1. Introduction

Urinary tract infections (UTIs) represent a common disorder treated by urologists and general medical practitioners, accounting for an important percentage of the yearly healthcare costs [1]. Most UTIs are treated on ambulatory patients [2]. However, the increasing resistance to the first-line antibiotic treatment [3,4,5] and the rising quota of this condition [6] have urged the research of new lines of therapy. In practice, we must combine updated data on uropathogens’ resistance profiles and sensibility rates of antimicrobial agents used in the treatment of UTIs. 

Several factors are linked to promoting the increasing spread of bacterial resistance to antibiotics in community settings. The most important vector of increasing resistance is represented by the overuse of antimicrobials in general medicine, veterinary, or agriculture, which enables the selection and spread of drug-resistant strains [7]. Other risk factors are host-related; an extensive review of the literature [8] aiming to detect the risk factors associated with multidrug-resistance (MDR) UTIs has highlighted 12 possible factors:

- Probable factors: urinary catheterization, previous hospitalization, previous antibiotic treatment, nursing home resident;
- Possible risk factor: age, previous UTI, male gender;
- Unlikely risk factors: diabetes, recent travel, ethnicity, immunocompromised, female gender.

International assemblies of heads of departments from international specialized forums such as the European Center for Disease Prevention and Control (Stockholm, Sweden), Office of Infectious Diseases, Department of Health and Human Services from Center for Disease Prevention and Control (Atlanta, GA, USA), and Division of Epidemiology, Tel Aviv Sourasky Medical Center (Tel Aviv, Israel) [9] proposed standard definitions for various categories of drug-resistant bacteria. These are classified as [9]:

- “multiple drug-resistant” (MDR)—nonsusceptible to one or more antibiotic agent in three or more antimicrobial categories,
- “extensive” or “extremely” drug-resistant (XDR)—nonsusceptible to one or more antibiotic agents in all but two or less antimicrobial classes, and
- “pan drug-resistant”(PDR)—nonsusceptible to all antimicrobial agents listed.

They admitted that a better understanding of highly resistant bacterial strains and obtaining comparability data would be facilitated if these definitions were applied worldwide. 

The European Association of Urology (EAU) via EAU Guidelines on Urological Infections from 2020 [10] recommends empirical treatment of uncomplicated urological infections using sulfonamides (TMP-SMX), phosphonic acids (fosfomycin), or nitrofurantoin. Fluoroquinolones (ciprofloxacin and levofloxacin) may be used only as an alternative therapy while also considering locoregional resistance rates. Carbapenems (imipenem and meropenem) should be used only as reserved therapy or in special conditions such as urosepsis. 

This study aimed to determine the resistance profiles of the most frequent multidrug-resistant uropathogen strains involved in UTIs on a Romanian male and female cohort. The preliminary data [11] have shown *Escherichia coli* as the most frequent bacteria (42.9%) implicated in UTIs, followed by *Klebsiella* spp. (21.17%), *Enterococcus* spp. (18.66%), *Proteus* spp. (7.75%), *Staphylococcus* spp. (4.91%), and *Pseudomonas aeruginosa* (4.58%). The limited number of MDR strains studied in previous research, the necessity of determining specific resistance patterns for each bacteria against common antibiotic classes in treating UTIs, and comparing the results with the international findings were the decisive factors in initiating the study.

## 2. Results

**Figure 1:**
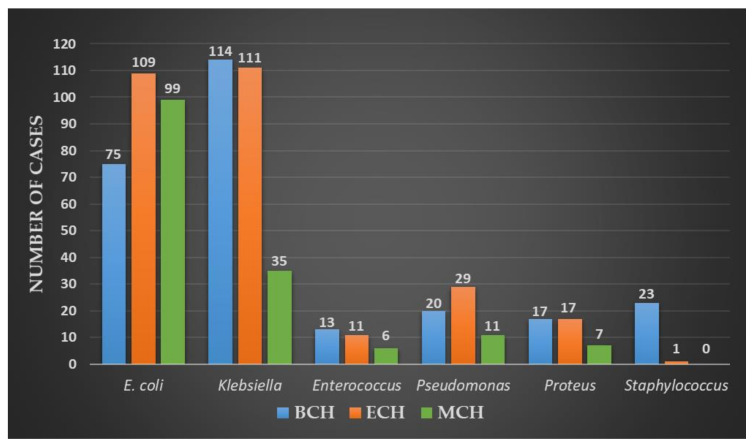
Distribution of the MDR uropathogens in the study centers.

A total number of 698 cases of MDR-UTIs were registered in all three centers during research, as follows: 262 patients (37.53%) at “Prof. Dr. Th Burghele” Clinical Hospital (BCH), 278 cases (39.82%) at Elias University Hospital (EUH), and 158 samples (22.63%) at Mures County Hospital (MCH). A detailed report on MDR-UTIs uropathogen distributions for each center is presented in Table 1 and Figure 1.

Except for BCH center, where *Klebsiella* spp. (43.51%) surpassed *E. coli* (28.62%) in prevalence, the rest of the Gram-negative uropathogens respected the distribution in all subjects. Overall, *Enterococcus* spp. was the most incriminated Gram-positive bacterial strain, except for the BCH center, where *Staphylococcus* spp. surpassed it in prevalence; in the EUH center, a single strain of MDR-*Staphylococcus* spp. was detected; as for the MCH center, none of these pathogens were registered.

In terms of age-group distribution, both males and females showed an increased prevalence at the lower pole of the distribution axis with 6.45% females and 6.68% males in their 18–29 years. We noted a progressive increase in MDR-UTIs with every decade of life. This highlights the correlation between age and incidence of UTIs; a high quota was observed in seniors between 60–69, representing 24.73% in females and 30.07% in males, with a peak of incidence in patients over 70 years old—48.28% in the overall population. Detailed data on age-group distribution is displayed in Table 2.

*E. coli*, the most frequent microbial strain, showed the highest resistance rate (R) to quinolones-levofloxacin—72.08%, followed by penicillin-ampicillin—66.78%, cephalosporins-ceftazidime—60.07%, and aminopenicillins + β lactamase-amoxicillin-clavulanate—56.89%. A good sensitivity (S) was observed for fosfomycin—83.74%, followed by amikacin—66.78% and nitrofurantoin—39%.

**Figure 2:**
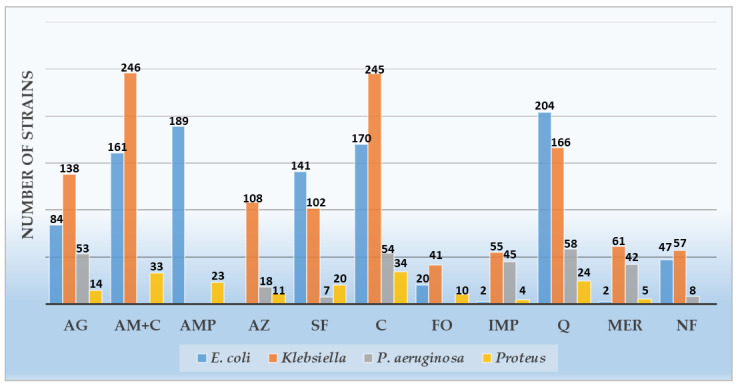
Gram-negative uropathogen resistance profiles (AG—aminoglycosides, AM + C—amoxicillin + clavulanic ac., AMP—ampicillin, AZ—aztreonam, SF—sulfonamides, C—cephalosporins, FO—Fosfomycin, IMP—imipenem, Q—quinolones, MER—meropenem, and NF—nitrofurantoin).

Detailed information of each Gram-negative bacterial strains, including resistance and sensitivity profiles and overall statistics, are included in Figure 2 and Table 3.

*Klebsiella* ranked as the second-most common uropathogen in the study. It showed an outstanding resistance to all antibiotic classes, led by amoxicillin-clavulanate—94.61%, followed by ceftazidime—94.23%, levofloxacin—63.84%, and amikacin—53.07%. No good sensitivity was observed for either of the tested antibiotics. None of them showed resistance below 10%. The lowest resistance was obtained for fosfomycin—15.75%, nitrofurantoin—21.92%, and carbapenems—imipenem and meropenem—21.15% and 23.46%, respectively.

The third-most frequent uropathogen, *P. aeruginosa*, shows almost complete resistance in MDR strains to quinolones-levofloxacin—96.66%; alarmingly, a high resistance is also observed for cephalosporins-ceftazidime—88.33%, followed by aminoglycosides-amikacin—88.33%. Surprisingly, the resistance to carbapenems in MDR *P. aeruginosa* is the highest in all pathogens for this antimicrobial class, accounting for imipenem—75% and meropenem—70%.

*Proteus* spp. is considered a nosocomial uropathogen, consistently discovered in patients presenting complicated UTIs. *Protea* (a group of pathogens including *Proteus*, *Providentia*, and *Morganella* spp.) are naturally resistant to colistin and nitrofurantoin and have raised resistance to carbapenems [12]. Our study discovered lower resistance rates than other Gram-negative bacteria, showing the highest resistance profile to ceftazidime—82.92%, followed by amoxicillin-clavulanate—80.48%, levofloxacin—58.53%, and ampicillin—56.09%. Relatively preserved sensitivity was observed for amikacin and meropenem—both 65.85%.

Both Gram-positive bacteria in this study make up for less than 10% of the total strains: *Staphylococcus* spp.—3.43% and *Enterococcus* spp.—4.29%; in both cases, the highest resistance was observed for quinolones (*Staphylococcus* spp.—91.66% and *Enterococcus* spp.—63.33%) and penicillin (*Staphylococcus* spp.—83.33% and *Enterococcus* spp.—70.0%).

The most frequent association of antimicrobial classes involved in common MDR strains was represented by amoxicillin-clavulanate, aztreonam, cephalosporins, and quinolones in 56 isolates (8.02%), followed by aminoglycosides, amoxicillin + clavulanate, sulfonamides, and cephalosporins in 5.01%; penicillin, sulfonamides, and quinolones in 4.87%; and aminoglycosides, amoxicillin + clavulanate, aztreonam, cephalosporins, carbapenems, and quinolones in 4.01%. Detailed outcomes of the 10th-most common MDR strain resistance patterns can be found in Table 4.

**Figure 3:**
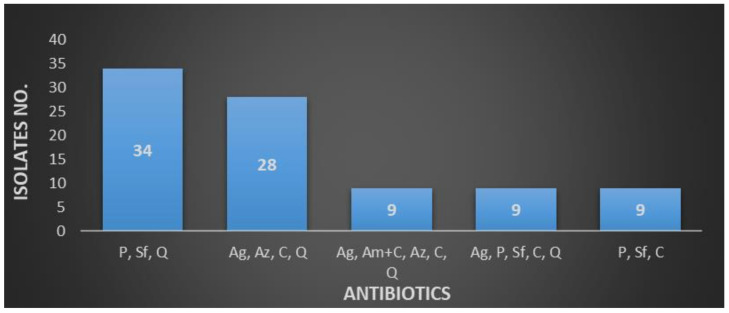
*Escherichia coli* resistance profiles of MDR strains (Ag—aminoglycosides, Am + C—aminopenicillins + β-lactams inhibitor, Az—aztreonam, C—cephalosporins, P—penicillin, Q—quinolones, and Sf—sulfonamides).

The resistance profile for *E. coli* and *Klebsiella* presented similarities, as well as noticeable differences, in the results. For *E. coli*, a high resistance to various combinations can be observed, such as penicillin, sulfonamides, and quinolones (*n* = 34 strains); aminoglycosides, aztreonam, cephalosporins, and quinolones (*n* = 28 strains); and aminoglycosides, aminopenicillins + β-lactams inhibitor, aztreonam, cephalosporins, and quinolones (*n* = 9 strains)—Figure 3.

**Figure 4:**
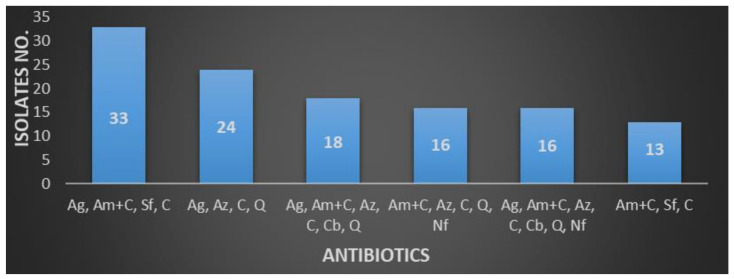
*Klebsiella* spp. resistance profiles of MDR strains (Ag—aminoglycosides, Am + C—aminopenicillins + β-lactams inhibitor, Az—aztreonam, C—cephalosporins, Cb—carbapenems, Nf—nitrofurantoin, Q—quinolones, and Sf—sulfonamides).

*Klebsiella* spp. revealed resistance to aminoglycosides, aminopenicillins + β-lactams inhibitor, sulfonamides, and cephalosporins (*n* = 33 strains), followed by aminoglycosides, aztreonam, cephalosporins, and quinolones (*n* = 24 strains) and aminoglycosides, aminopenicillins + β-lactams inhibitor, aztreonam, cephalosporins, carbapenems, and quinolones (*n* = 18 strains)—Figure 4. *E. coli* proved resistant mostly to penicillin and quinolones, while *Klebsiella* spp. to aminoglycosides, the aminopenicillins+ β-lactams inhibitor, or even carbapenems. 

*E. coli* proved resistant mostly to penicillin and quinolones, while *Klebsiella* spp. to aminoglycosides, the aminopenicillins+ β -lactams inhibitor, or even carbapenems. 

## 3. Discussion

Infections located in the urinary tract are a common cause of urological treatment among the general population. Moreover, acquiring a bacterial strain that shows resistance to multiple antimicrobial agents in use overlaps the primary morbidity of UTIs alone, leading to an excessively dangerous disease that in the absence of prompt and adequate treatment, can cause lots of problems. Due to overuse of antibiotics in various fields, such as general medicine, veterinary, or agriculture, an alarming increase of MDR strains among uropathogens was detected; taking into account the lack of locoregional data on MDR-UTIs incidence and resistance patterns, results from a six-month multicenter “cross-sectional” retrospective study are provided.

### 3.1. MDR Uropathogens in Relation with Patients Age

Throughout the entire cohort of patients, one could see a general trend of progressive growth in MDR-UTIs with every decade of life. This highlights the correlation between age and incidence of UTIs, just like Rowe et al. [13] summarized in a literature review. A high quota was observed in seniors between 60–69 years old, representing 24.73% in females and 30.07% in males, with a peak of incidence in patients over 70 years old—48.28% in the overall population. It has been shown that multiple risk factors are associated with the prevalence of UTIs in the elderly male population, such as prostate enlargement, urolithiasis, urinary tract neoplasia, renal failure, or urethral strictures [14,15]. 

In the elderly female population, various risk factors are associated with UTIs. The studies performed by Hu et al. [16], Brown et al. [17], and a literature review from Mody et al. [18] demonstrated that a history of UTIs during early lifetime, diabetes, functional disability, urinary retention, presence of urinary catheters, history of urogynecology surgery (all of them eventually combined with estrogen deficiency) are common risk factors-related with this age category. As an exception, a spike of incidence is underlined at the lower pole of the distribution axis; the increased number of positive MDR urine cultures in these patients is linked to the most active sexual period in both sexes, at the end of puberty, as Foxman B. [19] and Chu et al. [20] previously showed in their papers. It has been proven that high frequency of sexual intercourse, use of male condoms, contraceptive diaphragms, spermicides, and abusing some of the antimicrobials are key factors of acquiring UTIs at a younger age [21].

### 3.2. Comparison of MDR Escherichia coli Patterns with Other Studies

For the most common uropathogen in the tested cohort, high resistance rates to the most common antimicrobial agents were observed; similar results for MDR *E. coli* strains, with amoxicillin R = 55.6% and nitrofurantoin R = 7.9%, were obtained by Baral et al. [22] in 2012, while the results for ceftazidime and amikacin were R = 100% and R = 6.2%, respectively. Dehbanipour et al. [23] performed a study in Iran in 2016 that showed an alarming resistance in MDR *E. coli* to amikacin (R = 89.1%) higher than our findings and with an even higher difference compared to nitrofurantoin (R = 85.9%). The same paper admitted increasing resistance to carbapenems (meropenem), while we observed a still very low resistance in this group—R = 0.7%—both to imipenem and meropenem. 

In terms of MDR patterns, *E. coli* showed the highest one to penicillin and quinolones; the last one appeared in almost all MDR *E. coli* patterns. Linhares et al. [24] developed a similar study in 2015 in Aveiro, Portugal analyzing 4376 MDR uropathogens strains, searching for resistance patterns in MDR microorganisms; the report highlighted the highest resistance to the combination of penicillin, sulfonamides, and quinolones with 12.6%, followed by cephalosporins, nitrofurans, and penicillin with 7.8%; cephalosporins, penicillin, and sulfonamides in 6.6%; and cephalosporins, penicillin, quinolones, and sulfonamides with 6.0%. Contrary to the Portuguese research, the current findings suggest an increasing resistance for aminopenicillins and aminoglycosides in the tested MDR strains. A recent study from Iasi, Romania, conducted in early 2020 [25], obtained similar locoregional results, highlighting an increasing resistance to carbapenems. Surprisingly, the tested strains showed good sensitivity to fosfomycin (S = 83.74%); similar findings were reported by Sultan et al. in 2014 [26]. His study analyzed the resistance rates among uropathogens, especially MDR strains, highlighting 100% sensitivity to this drug from ESBL and non-ESBL *Enterobacteriaceae*, *Staphylococcus aureus*, and *Enterococcus fecalis*. 

### 3.3. Comparison of MDR Klebsiella Patterns with Other Studies

*Klebsiella* spp. is the Gram-negative bacteria ranked as the second-most frequent uropathogen in MDR-UTIs but the first in terms of resistance, as previously shown. Mishra et al. [27] published in 2013 extensive research on 996 MDR uropathogen strains sampled from hospitalized patients that were tested for common antibiotics in three different time phases. High rates of resistance were observed for *Klebsiella* spp. to multiple antibiotic classes. The Indian study highlighted higher resistance in 2013 compared to our results for amikacin (R = 65%, 76%, and 79%); trimethoprim-sulfamethoxazole (R = 66%, 69%, and 78%); and nitrofurantoin (R = 69%, 71%, and 76%), while, for amoxicillin-clavulanate (R = 54%, 67%, and 76%); ceftazidime (R = 64%, 69%, and 73%); and levofloxacin (R = 47%, 49%, and 53%), better sensitivity in all three phases was observed, contrary to our results. 

One can see that, for both *E. coli* and *Klebsiella* spp., the two most common uropathogens in MDR strains, an alarmingly increased resistance for beta-lactams and fluoroquinolones has been reported by many researchers. Various mechanisms of resistance—such as enzymatic inactivation, target modification, reduced permeability reduction, or active efflux, are cited for the beta-lactams [28]. For fluoroquinolones, chromosomal mutations altering target enzymes DNA gyrase and topoisomerase IV or activating the efflux systems and pumping drugs out of the cytoplasm, concomitant with the loss of porin channels for drug entry [29], are key factors in the resistance patterns. Numerous studies have highlighted the increasing resistance to beta-lactams (penicillin, aminopenicillins, and cephalosporins) and—even if numbers are still low—a continuous growth for carbapenems [30] for all uropathogens (especially for *E. coli* and *Klebsiella* spp. [31,32,33]). The same can be stated in regard to quinolones [34,35]. 

Thus, a careful administration for both antimicrobial classes is recommended, always considering the locoregional studies on antimicrobial resistance patterns. The European Guidelines on Urological Infections [10] also suggest similar cautiousness.

### 3.4. How MDR Strains Modify Our Daily Practice in UTIs

According to the European Association of Urology [10], the primal evaluation of a patient suspecting an UTI imposes an empiric antibiotic treatment, taking into account locoregional patterns of resistance, followed by the collection of urine specimens for uroculture; the antibiogram will later conduct the definitive treatment of the specific UTI. In suspected cases of MDR microorganisms, a careful decision on recommending an antibiotic should be made. 

A recent study from Germany conducted by Bischoff et al. [36] that was following the empiric antibiotic therapy in UTIs in patients with risk factors for resistant uropathogens noticed that, in patients with two or more risk factors, the lowest susceptibility was represented by quinolones-ciprofloxacin (S = 51.8%). A second-generation cephalosporin-cefuroxime (S = 53.7%) followed, then a third-generation cephalosporin-cefpodoxime (S = 60.7%), a wide broad-spectrum penicillin with a beta lactamase inhibitor-piperacillin/tazobactam (S = 75.0%), an aminoglycoside-gentamycin (S = 75.0%), another third-generation cephalosporin-ceftazidime (S = 76.8%), and the highest susceptibility was reported for carbapenems-imipenem (S = 91.1%). Another recent study from Italy published by Gasperini et al. [37] concerning MDR UTI bacteria in geriatric population also highlighted the increased uropathogen resistance for quinolones-levofloxacin (R = 80.0%) and ciprofloxacin (R = 55.3%); cephalosporins-cefepime, cefotaxime, and ceftazidime (R = 45.8%, 59.4%, and 51.6%, respectively); and aminopenicillin with beta lactamase inhibitor-amoxicillin/clavulanic acid (R = 50.0%) was noted. Favorable sensitivity patterns for aminoglycosides-amikacin (R = 9.4%) and carbapenems-meropenem (R = 8.5%) were also noted. 

All this data endorses the presented results, as this study observed the highest resistance for quinolones, cephalosporins, and aminopenicillins; intermediate resistance was noted for amikacin; and confined sensitivity was observed for carbapenems, although not enough ubiquitous testing in all three centers was conducted for this drug class. Surprisingly, promising sensibility was noted for fosfomycin; this “old forgotten drug”, as Bradley J Gardiner [38] referred to it in a study from early 2019 concerning the resistance patterns for nitrofurantoin and fosfomycin, could be an effective alternative for these patients, especially in uncomplicated UTIs.

Although fosfomycin is both a safe and effective drug in the management of the empiric treatment of a UTI, as MDR strains breed complicated features, its efficiency and usage must be limited to cystitis.

When a MDR uropathogen is suspected in complicated UTIs, the only viable treatment is represented by carbapenems.

### 3.5. Limitations

The impossibility of determining each bacterial species’ genus in all involved centers is the major limitation of the study. It would have been helpful if the minimal inhibitory concentration of each drug was measured, providing a better understanding of the resistance dynamics in each of the tested strains. The clinical information of every patient was not available, especially in the ambulatory patients, to allow a correlation between resistance profiles and risk factors, plus medical history. One of the study’s primary limitations was represented by the lack of assessment of the risk factors contributing to the emerging of multidrug resistance, resulting in the impossibility of suggesting specific recommendations. Considering that the study was performed only in tertiary-care hospitals where patients with more severe pathology are managed, this could represent a risk of selection bias. 

All the aforementioned would have improved the accuracy of the recommendations that would eventually reduce acquiring MDR bacteria.

## 4. Materials and Methods

### 4.1. Study Design and Sample Population

This descriptive “cross-sectional” retrospective study was conducted at three different clinical hospitals in two University centers: “Prof Dr. Th Burghele” Clinical Hospital (BCH) and Elias University Hospital (EUH) from Bucharest, Romania and Mures County Hospital (MCH) from Targu Mures, Romania. The data collection was conducted for six months, between 1 September 2018 and 28 February 2019.

**Figure 5:**
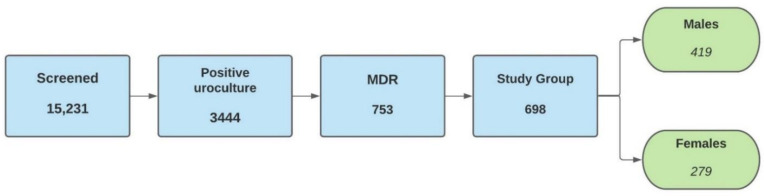
Diagram of the screened and enrolled patients in the study.

Urine probes from 15,231 patients were collected for bacterial analysis, out of which 3444 (22.61%) presented positive urine cultures with more than 10^5^ CFU/mL and 698 (4.58%) showed criteria of MDR-UTIs. The representative diagram of patient dynamics is illustrated in Figure 5.

Information on age, sex, and social–demographic status were registered for each patient; both hospitalized and ambulatory treated patients were considered for this study; thus, an exhaustive medical history for the second group was not available.

### 4.2. Inclusion and Exclusion Criteria

The inclusion criteria:

1. Positive urine culture ≥ 10^5^ CFU/mL;
2. Single bacteria strain on urine culture;
3. Age ≥ 18 years;
4. Standards of MDR-UTIs (nonsusceptible uropathogen to one or more antibiotic agents in three or more antimicrobial categories).

The exclusion criteria:

1. Urine culture < 10^5^ CFU/mL;
2. Two or more bacterial strains on uroculture;
3. No requirements of admission into the MDR group;
4. Presence of permanent urinary catheter (>1 month).

### 4.3. Sample Collection, Bacterial Culture, Identification of Uropathogens, and Antibiotic Susceptibility Test

The European and Romanian Association of Urology guidelines [10,39] on urological infections are followed to treat UTIs among patients from all clinics. In each case, a minimum 7–10 days were considered between the last antibiotic treatment and urine sampling for proper microbiological testing.

International Safety Standards [40] were followed for urine collecting techniques. After inoculation and incubation, microorganisms were identified based on specific Gram reactions, morphology, and biochemical characteristics. In all cases, the Clinical Laboratory Standard Institute (CLSI) [41] guidelines were followed for a comprehensive determination of sensitivity and resistance rates for each of the antibiotics tested to obtain the antibiogram.

Bacterial culture, the identification of uropathogens, and the antibiotic susceptibility test used were previously described in more detail [3,4,11].

### 4.4. Statistical Analysis

Data analysis was conducted using Microsoft Excel software (version 2020, Microsoft Corporation, Redmond, WA, USA); simple descriptive statistics were calculated. The relations of the variables were analyzed using frequency and percentage.

## 5. Conclusions

The current study acknowledged *E. coli* as the most common urinary pathogen among the MDR bacteria involved in UTIs among hospitalized and ambulatory patients. The incidence of MDR-UTIs increases with age distribution, peaking among the over 70 years old group of patients. In both the Gram-negative and Gram-positive groups, the highest resistance was noted for quinolones and β-lactams; alarmingly, the resistance to carbapenems rose, peaking with *P. aeruginosa*. Overall, the most common MDR resistance profiles were associated with aminopenicillins, quinolones, and cephalosporins. Together, *E. coli* and *Klebsiella* represented more than three-quarters of the identified MDR strains. 

As MDR in urological infections are evolving, we strongly recommend the surveillance of resistance profiles and assessing the risk factors. A proper antibiotic administration policy should be implemented considering new MDR resistance data entailing the locoregional results.

## Figures and Tables

**Table 1:** MDR isolated uropathogens.

Isolated Bacteria	BCH		EUH		MCH		Total	
	*n*	*%*	*n*	*%*	*n*	*%*	*n*	*%*
Gram negative	226	86.25	266	95.68	152	96.20	644	92.26
*Escherichia coli*	75	28.62	109	39.20	99	62.65	283	40.54
*Klebsiella* spp.	114	43.51	111	39.92	35	22.15	260	37.24
*Pseudomonas aeruginosa*	20	7.63	29	10.43	11	6.96	60	8.59
*Proteus* spp.	17	6.48	17	6.11	7	4.43	41	5.87
Gram positive	36	13.74	12	4.31	6	3.79	54	7.73
*Enterococcus* spp.	13	4.96	11	3.95	6	3.79	30	4.29
*Staphylococcus* spp.	23	8.77	1	0.35	-	-	24	3.43

*n*—number, %—percentage, BCH—Burghele Clinical Hospital, EUH—Elias University Hospital, and MCH—Mures County Hospital.

**Table 2:** Female and male age group distribution of the MDR uropathogens.

Age Groups (Years)	Females		Males		Total	
	*n*	*%*	*n*	*%*	*n*	*%*
18–29	18	6.45	28	6.68	46	6.59
30–39	14	5.01	9	2.14	23	3.29
40–49	16	5.73	15	3.57	31	4.44
50–59	33	11.82	33	7.87	66	9.45
60–69	69	24.73	126	30.07	195	27.93
≥70	129	46.23	208	49.64	337	48.28

*n*—number and %—percentage.

**Table 3:** Gram-negative uropathogen resistance profiles.

Antibiotics	Gram-Negative Organisms Isolated														
	*Escherichia coli*			*Klebsiella* spp.			*Pseudomonas aeruginosa*			*Proteus* spp.			Total		
	R	S	NA	R	S	NA	R	S	NA	R	S	NA	R	S	NA
	*n (%)*	*n (%)*	*n (%)*	*n (%)*	*n (%)*	*n (%)*	*n (%)*	*n (%)*	*n (%)*	*n (%)*	*n (%)*	*n (%)*	*n (%)*	*n %*	*n %*
Amikacin	84 (29.68)	189 (66.78)	10 (3.53)	138 (53.07)	120 (46.15)	2 (0.76)	53 (88.33)	6 (10.0)	1 (1.66)	14 (34.14)	27 (65.85)	-	289 (44.87)	342 (53.1)	13 (2.01)
Amoxicillin- Clavulanic ac.	161 (56.89)	114 (40.28)	8 (2.82)	246 (94.61)	11 (4.23)	3 (1.15)	-	-	-	33 (80.48)	7 (17.07)	1 (2.43)	440 (75.34)	132 (22.6)	12 (2.05)
Ampicillin	189 (66.78)	6 (2.12)	88 (31.09)	-	-	-	-	-	-	23 (56.09)	2 (4.87)	16 (39.02)	212 (65.43)	8 (2.46)	104 (32.09)
Aztreonam	-	-	-	108 (41.53)	3 (1.15)	149 (57.3)	18 (30.0)	2 (3.33)	40 (66.66)	11 (26.82)	6 (14.63)	24 (58.53)	137 (37.95)	11 (3.04)	213 (59.0)
Trimethoprim/ Sulfamethoxazole	141 (49.82)	58 (20.49)	84 (29.68)	102 (39.23)	35 (13.46)	123 (47.3)	7 (11.66)	1 (1.66)	52 (86.66)	20 (48.78)	4 (9.75)	17 (41.46)	270 (41.92)	98 (15.21)	276 (42.85)
Ceftazidime	170 (60.07)	101 (35.68)	12 (4.24)	245 (94.23)	14 (5.38)	1 (0.38)	54 (90.0)	6 (10.0)	0	34 (82.92)	6 (14.63)	1 (2.43)	503 (78.10)	127 (19.72)	14 (2.17)
Fosfomycin	20 (7.06)	237 (83.74)	26 (9.18)	41 (15.76)	60 (23.07)	159 (61.15)	-	-	-	10 (24.39)	6 (14.63)	25 (60.97)	71 (12.15)	303 (51.88)	210 (35.95)
Imipenem	2 (0.7)	81 (28.62)	200 (70.67)	55 (21.15)	136 (52.3)	69 (26.53)	45 (75.0)	6 (10.0)	9 (15.0)	4 (9.75)	12 (29.26)	25 (60.97)	106 (16.45)	235 (36.49)	303 (47.04)
Levofloxacin	204 (72.08)	47 (16.6)	32 (11.30)	166 (63.84)	68 (26.15)	26 (10.0)	58 (96.66)	1 (1.66)	1 (1.66)	24 (58.53)	10 (24.39)	7 (17.07)	452 (70.18)	126 (19.56)	66 (10.24)
Meropenem	2 (0.7)	86 (30.38)	195 (68.90)	61 (23.46)	145 (55.76)	54 (20.76)	42 (70.0)	10 (16.66)	8 (13.33)	5 (12.19)	27 (65.85)	9 (21.95)	110 (17.08)	268 (41.61)	266 (41.3)
Nitrofurantoin	47 (16.6)	111 (39.22)	125 (44.16)	57 (21.92)	38 (14.61)	165 (63.46)	8 (13.33)	5 (8.33)	47 (78.33)	-	-	-	112 (18.57)	154 (25.53)	337 (55.88)

*n*—number, %—percentage; R—resistant, S—sensitive, and NA—not available.

**Table 4:** Most common MDR profiles.

Antibiotics	*n* (%)
Amoxicillin + Clavulanate, Aztreonam, Cephalosporins, Quinolones	56 (8.02)
Aminoglycosides, Amoxicillin + Clavulanate, Sulfonamides, Cephalosporins	35 (5.01)
Penicillin, Sulfonamides, Quinolones	34 (4.87)
Aminoglycosides, Amoxicillin + Clavulanate, Aztreonam, Cephalosporins, Carbapenems, Quinolones	28 (4.01)
Amoxicillin + Clavulanate, Aztreonam, Cephalosporins, Quinolones, Nitrofurantoin	21 (3.0)
Aminoglycosides, Amoxicillin + Clavulanate, Aztreonam, Cephalosporins, Quinolones	19 (2.72)
Aminoglycosides, Penicillin, Cephalosporins	17 (2.43)
Aminoglycosides, Sulfonamides, Cephalosporins	14 (2.0)
Penicillin, Cephalosporins, Quinolones	12 (1.71)
Aminoglycosides, Cephalosporins, Carbapenems, Quinolones	11 (1.57)

*n*—number and **%**—percentage.

## Data Availability

Data supporting the reported results are available from the authors.
